# The role of social cognition in mental health trajectories from childhood to adolescence

**DOI:** 10.1007/s00787-023-02187-8

**Published:** 2023-03-31

**Authors:** Dimitris I. Tsomokos, Eirini Flouri

**Affiliations:** 1 School of Psychology and Neuroscience, University of Glasgow, Glasgow, Scotland UK; 2 Department of Psychology and Human Development, UCL Institute of Education, University College London, London, UK

**Keywords:** Theory of mind, False-belief attribution, Social competence, Social cognition, Mental health, Adolescence, Longitudinal study

## Abstract

We investigated the association between an aspect of Theory of Mind in childhood, false-belief understanding, and trajectories of internalising (emotional and peer) and externalising (conduct and hyperactivity) problems in childhood and adolescence. The sample was 8408 children from the UK’s Millennium Cohort Study, followed at ages 5, 7, 11, 14, and 17 years. Social cognitive abilities were measured at 5 and 7 years through a vignette version of the Sally–Anne task administered by an unfamiliar assessor in a socially demanding dyadic interaction. Internalising and externalising problems were measured via the Strengths and Difficulties Questionnaire at 7–17 years. Using latent growth modelling, and after controlling for sex, ethnicity, maternal education, verbal ability, and time-varying family income, we found that superior social cognitive abilities predicted a decrease in emotional problems over time. In sex-stratified analyses, they predicted decreasing conduct problem trajectories in females and lower levels of conduct problems at baseline in males.

## Introduction

Effective interpersonal relations rely on social cognitive abilities [31, 68] and are a core feature of adaptive functioning and social competence in childhood and adolescence [31, 51, 55], in turn inversely associated with psychopathology [9, 12, 20, 47, 53]. Early deficits in social competence can become evident in difficulties in both communication skills and social cognition [6, 83] as well as behavioural problems [82]. Deficits in social cognition specifically have been linked not only with later behavioural problems [23, 56], but also autism spectrum disorder and borderline personality disorder [77], attention-deficit / hyperactivity disorder [84], and social phobia [41]. In this study, we explore the role of a component of social cognition, Theory of Mind (ToM), in the trajectories of broad and specific mental health problems from middle childhood to late adolescence in the general population.

## Theory of Mind and social cognitive abilities

Theory of Mind (ToM) encompasses a complex set of socio-cognitive abilities [27] enabling us to navigate the social world [1] and communicate more efficiently [18, 24]. It entails ‘reading’ others’ minds [86] by inferring their mental states [57]. Brain regions implicated in ToM include the temporoparietal junction [61, 70], associated with belief attribution [71], the superior temporal sulcus, involved in mental state inference [28] and social perception [2], and the ventromedial prefrontal cortex [43, 76], involved in the regulation of negative emotion [35].

Several measures have been proposed to assess ToM as a single, well-defined construct [7], all in turn used to address two questions: When is ToM first established during typical development [33, 74, 80]? And do difficulties in ToM imply psychopathology [5, 77]? One of the earliest measures of ToM used storytelling with puppets, and established that most neurotypical children aged around 4–5 years can understand false beliefs [87]. A modified version using dolls, known as the Sally–Anne task (SAT), indicated that around 80% of the general population passed the test by age 5 [5]. However, during the last decade, this view of ToM has been challenged on several fronts [4, 32, 63, 72]. Heyes [34], for example, has provided evidence in favour of a ‘submentalising’ model, where ToM is the result of multiple independent social cognitive components working together. Two significant challenges were also identified [63]: different tests meant to measure distinct constructs actually track the same ToM construct (heterogeneity), while a single test meant to be measuring one construct can track multiple social cognitive abilities (lack of specificity). For example, the SAT is an elicited-response task demanding executive functions [29, 73], while it has been also established that the performance of children on this dyadic assessment depends on factors beyond false-belief understanding, as children closely monitor the conduct of their assessor and react to it, thereby employing other social cognitive skills to complete the task successfully [40]. Furthermore, the explicit attribution of false beliefs is closely related to language [19, 62], among other factors affecting individual differences in ToM [37].

## Poor social cognition and child psychopathology

The last decade has witnessed a renewed interest in the long-term effects of poor social cognition, with a 2022 systematic review synthesising the evidence from 12 longitudinal studies on the role of social communication in internalising and externalising problems [16]. Much of this evidence points to clear links between early deficits in social cognition and later internalising and externalising problems. For example, Oliver et al. [56] tracked conduct problems from age 4 to 13 years, identifying four trajectories (low problem levels, limited to childhood only, problems beginning in adolescence, early-onset persistent problems), and showed that all problem trajectory types, except the low problem type, were associated with social cognitive deficits. Miers et al. [52] found three groups during adolescence presenting social anxiety (high, varying; moderate, decreasing; low, decreasing), and provided evidence for an association between social skill deficits and interpersonal problems at school, especially in the case of moderate and high problem trajectories. In a large meta-analytic review, Trentacosta and Fine [82] established an association between early social cognition (in the form of emotion knowledge) and internalising and externalising problem trajectories with small to moderate effect sizes. Finally, in a recent study examining the developmental course of social cognitive skills rather than that of internalising and externalising problems, de la Osa et al. [17] tracked the trajectories of social cognitive abilities in a sample of 378 children from preschool to preadolescence (3–12 years) and found that preadolescents in the increasing social deficit trajectory presented with a higher level of interpersonal and behavioural problems at school.

## Aims of the study

In this work, we focus on the role of ToM and social cognitive abilities established at ages 5 and 7 years (middle childhood) in mental health trajectories from age 7 to age 17 years (late adolescence). To this end, we use data from the UK’s Millennium Cohort Study (MCS), a large longitudinal birth cohort that follows around 19,000 children born during 2000–2002 to explore the role of children’s performance on the SAT in their course of their mental health (internalising and externalising problems) until late adolescence. At 5 years old, the MCS children were administered a vignette version of the SAT by an unfamiliar interviewer, this being the first task among several cognitive assessments at that age [49]. The protocol had 11 pointing-and-talking interactions and 3 final questions for the child. The same protocol was implemented when the children were 7 years old. The number of children who answered all three questions correctly in both sweeps was much lower than expected, and the survey team attributed this to the change of assessment mode (using vignettes) and the delivery of the protocol (using it to build rapport) [49]. However, we consider these specific characteristics of the SAT as an opportunity to study a group of children who passed the test and thus demonstrated both (1) false-belief understanding and (2) above-average social interaction skills in a demanding social situation. We refer to the particular combination of these outcomes as ‘superior’ social cognition. The guiding research question here is whether ‘superior’ ToM and social cognition in childhood, as defined above in the context of the MCS surveys, are associated with mental health from middle childhood through to late adolescence.

In particular, our hypothesis is that superior ToM and social cognitive abilities established in childhood would predict better mental health over time, as measured in MCS at ages 7, 11, 14 and 17 years via the parent-reported Strengths and Difficulties Questionnaire (SDQ) [30], even after adjustment for confounders. Therefore, we controlled for sex, ethnicity, cognitive ability at baseline (age 5 years), time-varying family income, and parental education. In a further sex-stratified analysis, given the well-documented gender differences in internalising and externalising problems [42, 45, 88] and ToM [14, 22], we explored further whether superior social cognitive abilities predict SDQ trajectories differently based on sex.

## Methods

### Participants and analytic sample

**Fig. 1:**
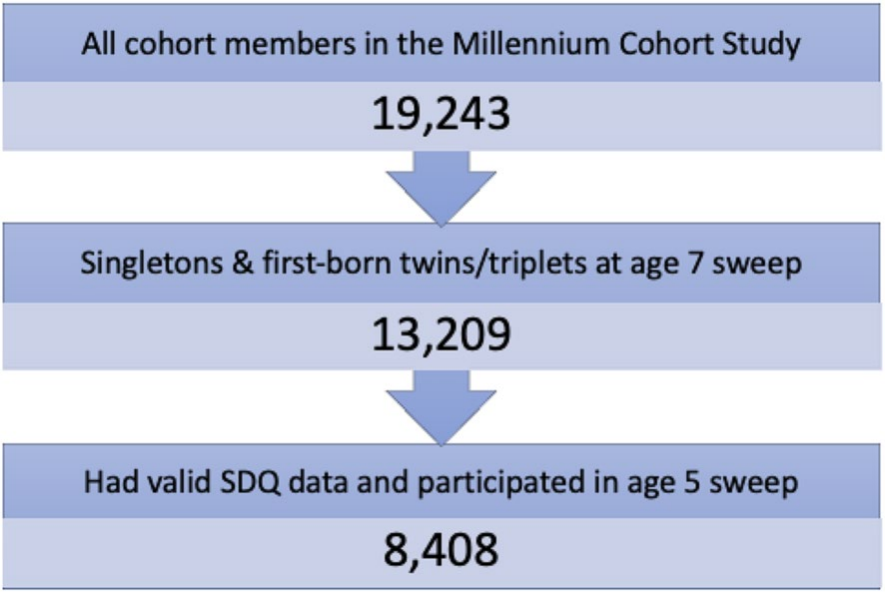
Sample selection process

MCS followed more than 19,000 children born in 2000–2002 [39], starting from around 9 months (sweep 1) to 3, 5, 7, 11, 14, and 17 years (sweeps 2 to 7, correspondingly). As explained by Plewis et al. [60], the sampling frame for MCS was provided by 338 electoral wards, and was designed to over-represent (a) families living in areas of high child poverty across the UK, (b) wards with high proportions of ethnic minorities in England, and (c) the smaller UK countries. Most of the information was collected through interviews with and self-completion questionnaires for the main adult respondent (overwhelmingly the mother) in the child’s home. Ethical approval was obtained from NHS Multi-Centre Ethics Committees, and all parents gave informed consent before interviews took place (the cohort children themselves gave their assent at age 11 years and their consent from age 14 years onwards). At the age 7 sweep, over 13,000 families took part. Our study’s analytic sample included cohort members that were singletons or first-born twins or triplets who (a) had valid data on the SDQ at age 7, and (b) had participated at both age 5 and age 7 sweeps, so that they had the chance to participate in the Sally–Anne task (SAT) assessment on both occasions. Of the 8,408 children (51% female) in the sample, all but 107 of them had complete data on the SAT at ages 5 and 7. Figure 1 shows the sample selection process.

### Measures and procedures

#### Mental health from childhood to adolescence (7–17 years)

The 25-point SDQ [30], with each item rated on a 3-point scale, was completed for the child by the parent (the mother in the vast majority of cases) at ages 7, 11, 14, and 17 years. The items combine into five broad scales: (1) emotional symptoms; (2) peer relationship problems; (3) prosocial behaviour; (4) conduct problems; and (5) hyperactivity/inattention. Each of these can take values from 1 to 11 in the MCS dataset. The first two scales constitute *internalising* (emotional) problems, and the last two scales correspond to *externalising* (behavioural) problems. Taken together (that is, without the prosocial scale) the four scales are used to calculate a total ‘difficulties’ SDQ score, which in the MCS dataset ranges from 1 to 41, after rescaling. As an established psychometric instrument [78], the SDQ has been shown to have good concurrent [54] and discriminant [46] validity, and is routinely used as a screening and assessment tool for mental health problems in this age group [48].

#### Sally–Anne task (SAT)

In this task, the child is introduced to two characters, Sally and Anne. Sally has a box, and Anne has a basket. Sally places a ball in her basket, and then leaves the room. In her absence, Anne takes the ball from the basket and moves it into the box. Children are asked to predict, on Sally's return to the room, (Q1) where Sally will look for the object (or, where she thinks the object is). In addition, children are asked two control questions: (Q2) a reality question (Where is the object, really?) and (Q3) a memory question (Where did Sally put the object at the beginning?) These three questions were asked at both age 5 and 7. In the present study, we require that a child had fully passed the test (Q1 to Q3 were answered correctly) in both interviews. Therefore, our predictor variable is whether a child had passed the SAT questions in both sweeps (therefore, the child’s SAT performance is given here in a dichotomous variable: ‘Passed’ or ‘Failed’).

#### Covariates

We adjusted for the following potential confounders. The family’s social background was approximated by the MCS sampling ‘*Stratum*’ (type of electoral ward within a UK country), which indexes the socioeconomic deprivation of each family’s area at the beginning of MCS. There are two strata in each country (England, Wales, Scotland, and Northern Ireland): advantaged and disadvantaged. In England, however, there is an additional, ‘*Ethnic minority*’, stratum, which includes wards that had an ethnic minority indicator of at least 30% in the 1991 Census, that is, at least 30% of their total population fell into the two categories ‘Black’ (Black Caribbean, Black African and Black Other) or ‘Asian’ (Indian, Pakistani, and Bangladeshi). The ‘*Disadvantaged'* stratum in England includes wards which were not part of the ethnic minority stratum, and which fell into the upper quartile (poorest 25% of wards) of the ward-based Child Poverty Index (CPI). Finally, the *‘Advantaged'* stratum includes wards which were neither a part of the ethnic minority stratum nor in the top quartile of the CPI. Maternal education (‘*Mat Edu*’) was the educational level of the main respondent attained by the age 5 sweep. This is an interval variable, ranging from 1 (no qualifications) to 6 (corresponding to the UK’s National Vocational Qualifications Level 5). In terms of individual characteristics, ‘*Ethnicity*’ is a covariate with 6 possible values, derived from the main respondent questionnaire at the age 5 sweep: White, mixed, Indian, Pakistani and Bangladeshi, Black or Black British, other ethnic group (including Chinese or other). ‘*Sex*’ (male/female) is a binary variable as reported by the main respondent. We have also considered the child’s expressive language ability (‘*Verbal ability’*) as assessed at age 5 (with values that range from 20–80) with a picture-naming cognitive test (ability and age adjusted based on British Ability Scales II age-normed data). Finally, ‘*income*’ is a household-level covariate, given in OECD equivalised income quintiles. It is tracked on every sweep in the MCS, and we treat it as a time-dependent variable. As any change in family income arguably takes time to influence mental health outcomes [79], we follow a time-lagged approach and consider the influence of family income from sweep *k* on SDQ measures at sweep *k* + *1*.

### Analytic strategy

#### Sample bias and missing data

Sample bias analysis was performed using unweighted descriptive statistics to identify the profile of our analytic sample in comparison to the non-analytic sample (‘rest of MCS’) at age 7 years. The volume of missing data was also identified at this stage, and this informed the imputation process.

#### Difference of means and correlations

The difference of SDQ means between the two groups for SAT (those children who passed the SAT and those who did not) was tested for independence in order to establish a main effect for SDQ at age 7, 11, 14, and 17 yeas. We also calculated the (unweighted) pairwise correlations between SDQ and the continuous numerical covariates, namely, income, maternal education, and verbal ability.

#### Latent growth model (LGM)

**Fig. 2:**
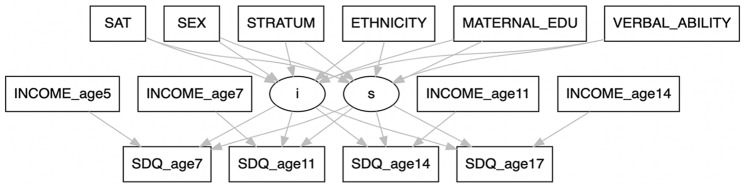
The hypothesised latent growth model. *Note* Rectangles represent observed variables, circles represent latent variables, and arrows denote associations. The latent variables, *i* and *s*, denote the LGM’s intercept (mean values of SDQ scales at baseline, age 7 sweep) and slope (rate of change of SDQ mean values from age 7 to age 17 sweep), respectively

Latent growth curve modelling [25] is a powerful tool in longitudinal research for tracking changes over time [11]. We use it here to understand the role of ToM in SDQ (a) at the starting point (baseline) at age 7 and (b) over time (across ages 7, 11, 14, and 17 years). Taking into consideration the covariates described previously, we formulate a structural equation model as depicted in Fig. 2.

To examine the link between passing the false-belief task in childhood (‘*SAT*’) and the overall growth of mental health problems (‘*total SDQ*’) from childhood to adolescence, that is, for $t\in [\mathrm{1,4}]$ survey sweeps, we fitted 3 LGMs, as explained below. We follow the latest best practices [58] and assess model fit using the robust standardised root mean squared residual (SRMR) for each of our models, considering a good fit only when SRMR < 0.08, based on accepted recommendations [36]. We started from a core, minimally adjusted, model for each cohort member in the sample (represented by $m\in [1, 8408])$, with only sex and stratum as covariates.

1 $$SD{Q}_{m}={a}_{m}+ {b}_{1,m}\times SAT+{b}_{2,m}\times sex+{b}_{3,m}\times stratum.$$

We adjusted this core model by adding ethnicity, level of maternal education, and the cohort member’s standardised verbal ability score (at age 5 sweep):

2 $$SD{Q}_{m}=model \left(1\right)+ {b}_{4,m}\times ethnicity+{b}_{5,m}\times Mat Edu + {b}_{6,m}\times verbal.$$

Finally, we added to the adjusted model (2) the time-varying family income:

3 $$SD{Q}_{m} = model \left(2\right)+{b}_{7,m}\times Income\left(t\right).$$

#### Supplementary analysis by sex

In an additional step, we stratified our analysis by sex. We fitted only the fully adjusted LGM (3) without the sex covariate on the four subscales for internalising and externalising problems as well as on the total SDQ scale.

#### Imputation process and data analysis

Missing data on all the covariates were imputed using multiple imputation by chained equations (MICE) for mixed data, on the assumption that they were missing at random [65]. We generated 100 imputed datasets based on the classification and regression trees (CART) algorithm, also known as decision trees [13], and used Rubin’s combination rules to consolidate the obtained individual estimates into a single set of multiply imputed estimates [67]. All numerical calculations were performed using R (R.Core.Team, 2021) with the ‘*mice*’ package and the ‘*cart’* method [85], while ‘*lavaan.survey*’ was used as a convenient wrapper for the ‘l*avaan*’ package for structural equation modelling [66]. For reproducibility, we note that the random seed was set to 357, and imputation was performed on our dataframe ‘*df*’ via the command: *mice(df, m* = *100, seed* = *357, method* = *"cart"*) to obtain the survey design with an imputation list (‘*df_survey’*) prior to fitting the latent growth model [*fit* <—*growth(model, data* = *df)*] and [*output* <—*lavaan.survey(fit, df_survey*)]. Our findings were reproduced and checked for convergence with a different random seed (123) and increasing imputation numbers (25, 50, 75, 100).

## Results

### Sample bias

Compared to the rest of MCS at age 7 sweep, our analytic sample was slightly over-indexed in girls, children of White ethnic background, and those from less disadvantaged areas. Income was moderately higher (Cohen’s *d* = 0.40) as were maternal education (*d* = 0.46) and verbal ability (*d* = 0.32), as seen in Table 1.

**Table 1:** Sample bias: variable distribution differences between the analytic sample and the rest of the MCS at age 7 sweep (unweighted)

	Rest of MCS *n* = 4801 (36%)	Analytic sample *n* = 8408 (64%)	Statistic	*p*
Categorical variables, *n* (%)				
SAT				
Pass	266 (6%)	595 (7%)	9.19	0.002
Fail	4350 (91%)	7706 (92%)		
Sex				
Female	2228 (46%)	4279 (51%)	24.42	< 0.001
England – Adv	1095 (23%)	2594 (31%)	169.42	< 0.001
Stratum				
England – Disadv	1209 (25%)	1993 (24%)		
England – Ethnic	644 (13%)	888 (11%)		
Wales – Adv	180 (4%)	413 (5%)		
Wales – Disadv	550 (12%)	771 (9%)		
Scotland – Adv	266 (6%)	533 (6%)		
Scotland – Disadv	347 (7%)	402 (5%)		
N. Ireland – Adv	173 (4%)	350 (4%)		
N. Ireland – Disadv	337 (7%)	464 (6%)		
Ethnicity				
Mixed	125 (3%)	225 (3%)		
Indian	101 (2%)	226 (3%)		
Pakistani and Bangladeshi	326 (7%)	477 (6%)		
Black or Black British	199 (4%)	211 (3%)		
Other ethnic group	80 (2%)	101 (1%)		
Continuous variables, mean (sd)				
Mat edu (min 1, max 6)	3.14 (1.44)	3.79 (1.38)	− 24.27	< 0.001
Verbal ability (min 20, max 80)	51.30 (12.10)	55.05 (11.43)	− 17.39	< 0.001
Income-age7 (min 1, max 5)	2.64 (1.37)	3.19 (1.39)	− 21.76	< 0.001

Statistic and *p* values for categorical and continuous variables correspond to Pearson’s ${\upchi }^{2}$ tests with Yates’ continuity correction and Welch’s two-sample *t* tests, respectively. ‘Adv.’ (‘Disadv.’) stands for advantaged (disadvantaged); and (min *i*, max *j*) denotes the range between the minimum and maximum values

### Missing values

The analytic sample of 8408 children was made up of MCS cohort members who were present at both age 5 and age 7 sweeps, and who had complete SDQ data at age 7. However, for subsequent sweeps, the SDQ variables had missing values of around 10.8% at age 17, 2.7% at age 14, and 2.5% at age 11. The ‘SAT’ variable had only 107 (1.3%) of its values missing. Maternal education had 3.7%, verbal ability had 1%, while income variables in different sweeps had between 3.1% and 8.9% missing values. There was zero missingness in sex and stratum, and only 2 (0.02%) values were missing for ethnicity.

### Difference of means and correlations

**Fig. 3:**
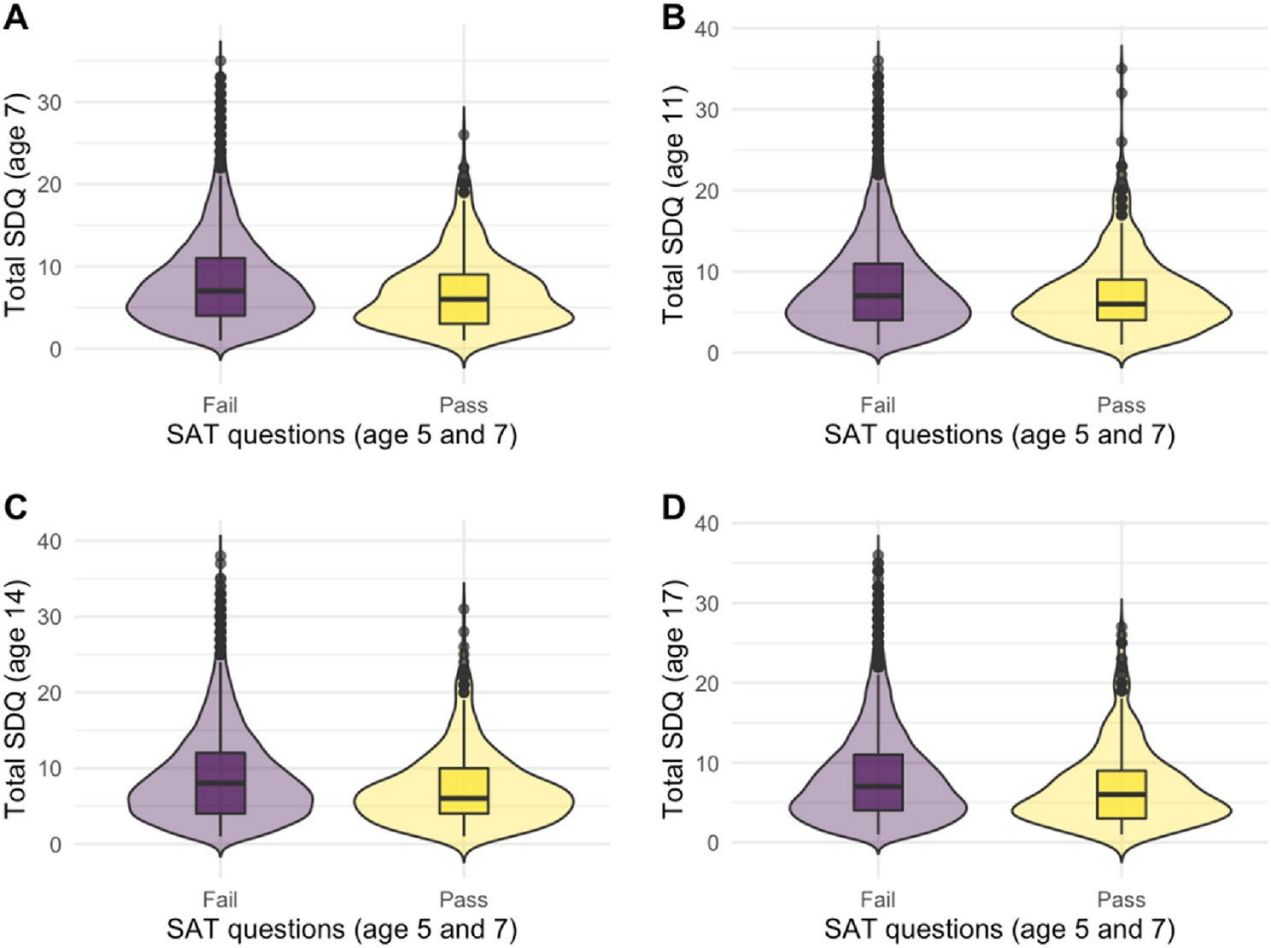
Violin box plots of unweighted total SDQ scores for Pass and Fail groups in SAT across sweeps: age 7 (subplot A), age 11 (**B**), age 14 (**C**), and age 17 (**D**)

Scores on the outcome variable (total ‘*SDQ*’) at baseline (age 7 sweep) were lower (*M* = 6.87, *SD* = 4.41) for those who had passed the SAT compared to those who had not (*M* = 8.10, *SD* = 5.22), where *t*(728.99)  = 6.468, *p* < 0.001, Cohen’s *d* = 0.25, 95% CI = [0.18, 0.33]. The same holds true for the remaining survey sweeps in our study, with an effect size of *d* = 0.22, 95% CI = [0.14, 0.30] at age 11, *d* = 0.27, 95% CI = [0.19, 0.35] at age 14, and *d* = 0.24, 95% CI = [0.16, 0.32] at age 17. Figure 3 depicts the differences with additional visual information included in violin box plots.

**Fig. 4:**
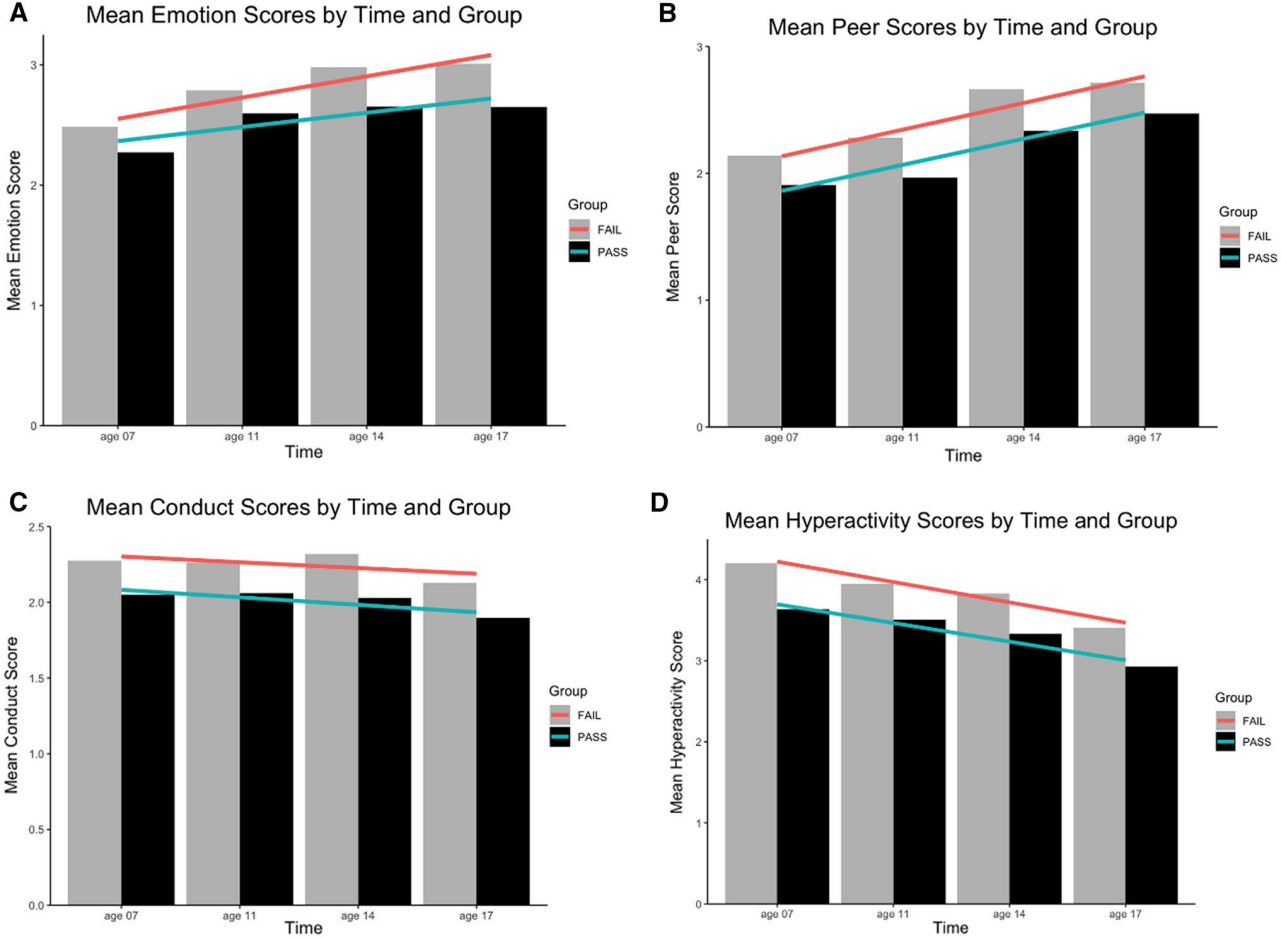
Bar charts of unweighted SDQ subscales for Pass (grey) and Fail (black) on SAT across sweeps: (**A**) emotion, (**B**) peer, (**C**) conduct, and (**D**) hyperactivity scores

To complete the bivariate analysis and depict the change over time (from age 7 to 11, 14, and 17 years), we present the results for each subscale for (A) emotional, (B) peer, (C) conduct, and (D) hyperactivity problems against pass–fail values for SAT in Fig. 4. We find that, in our analytic sample, (A) emotional problems *increase* over time for all from *M* = 2.47 (SD = 1.72) at age 7 years to 2.98 (2.24) at age 17 years; (B) peer problems *increase* from 2.12 (1.48) to 2.70 (1.77); (C) conduct problems *decrease* from 2.26 (1.45) to 2.11 (1.45); and (D) hyperactivity problems *decrease* from 4.16 (2.44) to 3.37 (2.21). For all the subscales, mean scores for those in the ‘Pass’ group (black bars in Fig. 4) are, in every age group, below those in the ‘Fail’ group (grey bars). In addition, emotional problems increase more for the ‘Fail’ group (red line in subplot A) compared to those in the ‘Pass’ group (green line).

We also calculated the pairwise correlations between the dependent variable (total SDQ) [at both baseline (age 7) and endpoint (age 17)] and the numerical covariates (income at age 5, verbal ability, and maternal education), as in Table 2. Correlation strength was weak to moderate, with the strongest association found for family income and maternal education ($r=.51,\mathrm{ t}(7824)=52.18, 95\mathrm{\% CI}=[.49, .52], p<.001$). We note that, in each survey sweep, the total difficulties SDQ scale had acceptable internal consistency: Cronbach’s $\alpha =.71$ (age 7); $\alpha =.73$ (age 11); $\alpha =.73$ (age 14); and $\alpha =.73$ (age 17 years). The consistency of all the subscales improved over time; however, at baseline (age 7), the subscales for peer ($\alpha =.51$) and conduct ($\alpha =.57$) problems had poor consistency, whereas those for emotional ($\alpha =.62$) and hyperactivity ($\alpha =.75$) problems had better consistency.

**Table 2:** Correlations between the total SDQ at ages 7 and 17 years and the continuous covariates at baseline

Variable 1	Variable 2	*r*	*p*	df error
Mat Edu	Income_age5	0.508	< 0.001	7824
Verbal	Mat Edu	0.321	< 0.001	8091
Verbal	Income_age5	0.308	< 0.001	8029
Verbal	SDQ_age17	− 0.176	< 0.001	7414
Mat Edu	SDQ_age17	− 0.212	< 0.001	7225
Verbal	SDQ_age7	− 0.216	< 0.001	8317
Income_age5	SDQ_age17	− 0.225	< 0.001	7240
Mat Edu	SDQ_age7	− 0.230	< 0.001	8091
Income_age5	SDQ_age7	− 0.257	< 0.001	8116

Pearson’s correlation reported, with Bonferroni correction applied to the Welch *t* tests for *p* values

### Latent growth models for total SDQ

In all three LGMs (weighted, imputed), having passed the SAT predicted decreasing trajectories (negative slope). Even in the fully adjusted case (3), ‘Pass’ was a significant predictor of decreasing SDQ over time (${b}_{1}=$− $0.171, se=0.077, z=$− $2.216, p=.027$). The covariances between the LGM intercept and slope are related in all models (for instance, in model 3 we have $Cov\left(i,s\right)=$ − $1.562, se=0.187, z=$− $8.371, p<.001$). All our models have a robust SRMR of up to 0.022, or less, which indicates a very good fit for a structural equation model. Tables 3, 4 include the regression coefficient estimates for the LGM slope and intercept, respectively. We note that, even in the fully adjusted model, males start out with higher total SDQ scores at baseline, as expected (${a}_{2}=1.293, se=0.129, z=10.048, p<.001$), compared to females, but they decrease over time (${b}_{2}=-0.432, se=0.053, z=-8.199, p<.001$).

**Table 3:** LGM slopes corresponding to regression models 1, 2, and 3 for total SDQ scores from age 7 to age 17 sweeps (imputed, weighted data)

	Model 1	Model 2	Model 3
Slope	Estimate	Estimate	Estimate
	(se)	(se)	(se)
SAT: pass	− 0.171*	− 0.161*	− 0.171*
	(0.077)	(0.077)	(0.077)
Sex: male	− 0.426***	− 0.427***	− 0.432***
	(0.053)	(0.053)	(0.053)
Stratum: England—disadvantaged	0.014	0.024	− 0.019
	(0.058)	(0.062)	(0.066)
England—Ethnic	− 0.296**	− 0.103	− 0.136
	(0.101)	(0.130)	(0.133)
Northern Ireland—advantaged	0.081	0.071	0.034
	(0.105)	(0.105)	(0.107)
Northern Ireland—disadvantaged	− 0.049	− 0.081	− 0.161
	(0.109)	(0.112)	(0.118)
Scotland—advantaged	0.075	0.067	0.072
	(0.092)	(0.092)	(0.092)
Scotland—disadvantaged	0.074	0.058	0.044
	(0.103)	(0.104)	(0.107)
Wales—advantaged	0.135	0.124	0.115
	(0.124)	(0.124)	(0.125)
Wales—disadvantaged	0.039	0.017	− 0.031
	(0.065)	(0.075)	(0.076)
Ethnicity: Black		− 0.072	− 0.092
		(0.153)	(0.154)
Indian		− 0.468*	− 0.456*
		(0.196)	(0.196)
Mixed		− 0.081	− 0.069
		(0.132)	(0.132)
Other ethnic (inc Chinese, other)		− 0.066	− 0.042
		(0.324)	(0.324)
Pakistani and Bangladeshi		− 0.349*	− 0.422*
		(0.168)	(0.170)
Maternal education		− 0.019	− 0.011
		(0.019)	(0.021)
Verbal ability		− 0.001	− 0.001
		(0.003)	(0.003)
Income-Age5			− 0.301***
			(0.041)
Robust SRMR	0.020	0.014	0.022
Number of observations	8408	8408	8408

***p < 0.001; **p < 0.01; *p < 0.05 | *SRMR* standardised root mean square residual

**Table 4:** LGM intercepts corresponding to regression models 1, 2, and 3 for total SDQ scores from age 7 to age 17 sweeps (imputed, weighted data)

	Model 1	Model 2	Model 3
Intercept	Estimate	Estimate	Estimate
	(se)	(se)	(se)
SAT: pass	− 0.836**	− 0.392	− 0.358
	(0.242)	(0.247)	(0.247)
Sex: male	1.213***	1.275***	1.293***
	(0.129)	(0.129)	(0.129)
Stratum: England—disadvantaged	1.621***	0.986***	0.846***
	(0.193)	(0.208)	(0.209)
England—ethnic	2.230***	1.071	0.898
	(0.600)	(0.585)	(0.586)
Northern Ireland—advantaged	− 0.704*	− 0.570	− 0.655*
	(0.296)	(0.296)	(0.297)
Northern Ireland—disadvantaged	1.566***	0.715	0.488
	(0.381)	(0.387)	(0.390)
Scotland—advantaged	− 0.220	− 0.051	− 0.088
	(0.313)	(0.313)	(0.313)
Scotland—disadvantaged	0.842*	0.348	0.184
	(0.373)	(0.376)	(0.376)
Wales—advantaged	0.115	0.018	− 0.024
	(0.320)	(0.321)	(0.321)
Wales—disadvantaged	1.351***	0.486	0.294
	(0.263)	(0.274)	(0.277)
Ethnicity: Black		− 0.995*	− 1.111*
		(0.469)	(0.472)
Indian		− 0.306	− 0.302
		(0.512)	(0.513)
Mixed		0.039	− 0.048
		(0.386)	(0.386)
Other ethnic (inc Chinese, other)		− 0.863	− 0.950
		(0.658)	(0.659)
Pakistani and Bangladeshi		− 0.255	− 0.366
		(0.520)	(0.522)
Maternal education		− 0.649***	− 0.535***
		(0.054)	(0.057)
Verbal ability		− 0.062***	− 0.058***
		(0.008)	(0.008)
Income-Age5			− 0.301***
			(0.041)
Robust SRMR	0.020	0.014	0.022
Number of observations	8408	8408	8408

***p < 0.001; **p < 0.01; *p < 0.05 | *SRMR* standardised root mean square residual

### Latent growth model for internalising and externalising subscales

The fully adjusted LGM (weighted, imputed) was fitted for each of the SDQ subscales of emotional/peer (internalising) and conduct/hyperactivity (externalising) problems. The SAT-pass group had decreasing trajectories (negative slope) for emotional problems (${b}_{1}=$− $0.089, se=0.034, z=$− $2.594, p=.009$). Tables 5, 6 include the regression coefficient estimates for the LGM slope and intercept, respectively. Here too, we found that males start out with higher internalising and externalising problems compared to females, with hyperactivity scores showing the greatest difference (${a}_{2}=0.866, se=0.059, z=14.591, p<.001$), decreasing over time (${b}_{2}=$− $0.064, se=0.023, z=$− $2.788, p=.005$).

**Table 5:** LGM slopes corresponding to the fully adjusted model (3) for the SDQ subscales from age 7 to age 17 sweeps (imputed, weighted data)

	Emotion	Peer	Conduct	Hyper
Slopes	Estimate (se)			
SAT: pass	− 0.089**	− 0.022	− 0.025	− 0.043
	(0.034)	(0.027)	(0.022)	(0.037)
Sex: male	− 0.275***	− 0.034*	− 0.065***	− 0.064**
	(0.020)	(0.015)	(0.014)	(0.023)
Stratum: England—disadvantaged	− 0.012	– 0.007	0.008	0.016
	(0.027)	(0.023)	(0.018)	(0.028)
England—ethnic	− 0.058	− 0.029	− 0.044	− 0.026
	(0.050)	(0.043)	(0.034)	(0.059)
Northern Ireland—advantaged	− 0.059	− 0.027	0.084**	0.034
	(0.041)	(0.031)	(0.032)	(0.055)
Northern Ireland—disadvantaged	− 0.009	− 0.102**	− 0.019	− 0.055
	(0.056)	(0.038)	(0.031)	(0.049)
Scotland—advantaged	0.065	0.006	0.011	− 0.004
	(0.037)	(0.036)	(0.026)	(0.038)
Scotland—disadvantaged	0.057	− 0.015	− 0.007	− 0.001
	(0.044)	(0.039)	(0.036)	(0.047)
Wales—advantaged	0.035	0.065	0.029	− 0.026
	(0.050)	(0.034)	(0.034)	(0.052)
Wales—disadvantaged	-0.014	-0.026	0.001	− 0.005
	(0.038)	(0.029)	(0.025)	(0.036)
Ethnicity: Black	− 0.089	− 0.101	0.081	0.012
	(0.069)	(0.054)	(0.042)	(0.062)
Indian	− 0.112*	− 0.206**	0.038	− 0.138
	(0.055)	(0.067)	(0.057)	(0.086)
Mixed	− 0.074	− 0.102*	0.021	0.085
	(0.055)	(0.047)	(0.042)	(0.062)
Other ethnic (inc Chinese, other)	− 0.129	− 0.103	0.135	0.082
	(0.135)	(0.076)	(0.069)	(0.121)
Pakistani and Bangladeshi	− 0.202**	− 0.253***	0.102*	− 0.059
	(0.068)	(0.065)	(0.047)	(0.064)
Maternal education	− 0.004	− 0.004	− 0.005	0.009
	(0.009)	(0.007)	(0.006)	(0.010)
Verbal ability	− 0.001	0.001	0.001	0.001
	(0.001)	(0.001)	(0.001)	(0.001)
Income-Age5	− 0.086***	− 0.069***	− 0.105***	− 0.102***
	(0.016)	(0.014)	(0.013)	(0.020)
Robust SRMR	0.014	0.016	0.020	0.014
Number of observations	8408	8408	8408	8408

***p < 0.001; **p < 0.01; *p < 0.05 |* SRMR* standardised root mean square residual

**Table 6:** LGM intercepts corresponding to the fully adjusted model (3) for the SDQ subscales from age 7 to age 17 sweeps (imputed, weighted data)

	Emotion	Peer	Conduct	Hyper
Intercepts	Estimate (se)			
SAT: pass	− 0.016	− 0.076	− 0.088	− 0.156
	(0.086)	(0.075)	(0.065)	(0.109)
Sex: male	− 0.047	0.195***	0.288***	0.866***
	(0.043)	(0.037)	(0.035)	(0.059)
Stratum: England—disadvantaged	0.143*	0.214***	0.210***	0.266**
	(0.058)	(0.056)	(0.052)	(0.095)
England—ethnic	0.172	0.234	0.228	0.236
	(0.166)	(0.160)	(0.121)	(0.208)
Northern Ireland—advantaged	− 0.102	− 0.186*	− 0.191**	− 0.208
	(0.080)	(0.078)	(0.069)	(0.149)
Northern Ireland—disadvantaged	− 0.021	0.068	0.205*	0.196
	(0.122)	(0.107)	(0.104)	(0.149)
Scotland—advantaged	− 0.115	− 0.085	0.095	0.005
	(0.099)	(0.073)	(0.080)	(0.138)
Scotland—disadvantaged	− 0.077	− 0.008	0.066	0.176
	(0.119)	(0.090)	(0.109)	(0.164)
Wales—advantaged	0.034	− 0.187	0.016	0.119
	(0.100)	(0.107)	(0.084)	(0.136)
Wales—disadvantaged	0.002	0.051	0.100	0.096
	(0.082)	(0.075)	(0.075)	(0.105)
Ethnicity: Black	− 0.194	0.022	− 0.365**	− 0.596**
	(0.188)	(0.124)	(0.130)	(0.223)
Indian	− 0.218	0.194	− 0.180	− 0.145
	(0.144)	(0.140)	(0.136)	(0.249)
Mixed	0.091	0.193	− 0.130	− 0.217
	(0.130)	(0.114)	(0.116)	(0.161)
Other ethnic	0.093	− 0.010	− 0.438**	− 0.643
	(0.244)	(0.165)	(0.156)	(0.337)
Pakistani and Bangladeshi	0.073	0.340	− 0.422***	− 0.431*
	(0.195)	(0.184)	(0.075)	(0.179)
Maternal education	− 0.096***	− 0.096***	− 0.123***	− 0.201***
	(0.019)	(0.016)	(0.017)	(0.026)
Verbal ability	− 0.013***	− 0.008***	− 0.010***	− 0.026***
	(0.003)	(0.002)	(0.002)	(0.003)
Income-Age5	− 0.086***	− 0.069***	− 0.105***	− 0.102***
	(0.016)	(0.014)	(0.013)	(0.020)
Robust SRMR	0.014	0.016	0.020	0.014
Number of observations	8408	8408	8408	8408

***p < 0.001; **p < 0.01; *p < 0.05 |* SRMR* standardised root mean square residual

### Supplementary sex-stratified analysis

The fully adjusted LGM (3)—without the sex covariate—was fitted for the SDQ subscales of internalising and externalising problems stratified by sex. In the case of boys (4129 cohort members, with 6% SAT ‘Pass’), we found that passing the SAT predicted fewer conduct problems at baseline ($${a}_{1}=-0.202, se=0.080, z=-2.510, p=.012)$$. In the case of girls (4279 cohort members, with 8% SAT ‘Pass’), it predicted a negative slope in the conduct problems’ trajectory ($${b}_{1}=-0.058, se=0.027, z=-2.184, p=.029)$$. Results of the LGM (3) for the SDQ subscales, and for the total SDQ as well, are presented in Table 7.

**Table 7:** LGM slopes and intercepts by sex for SAT: ‘Pass’ in LGM (3) for the SDQ subscales and the total SDQ from age 7 to age 17 sweeps (imputed, weighted data)

	Slope for boys	Intercept for boys	Slope for girls	Intercept for girls
Scale	Estimate			
	(se)			
Emotion	− 0.078	− 0.103	− 0.100	0.040
	(0.046)	(0.145)	(0.052)	(0.131)
Peer	− 0.045	− 0.062	− 0.005	− 0.087
	(0.042)	(0.122)	(0.040)	(0.100)
Conduct	0.018	− 0.202*	− 0.058*	− 0.008
	(0.036)	(0.080)	(0.027)	(0.089)
Hyperactivity	− 0.061	− 0.148	− 0.026	− 0.168
	(0.064)	(0.159)	(0.054)	(0.143)
Total SDQ	− 0.147	− 0.550	− 0.187	− 0.234
	(0.125)	(0.355)	(0.112)	(0.347)
Robust SRMR (max)	0.023	0.023	0.022	0.022
Number of observations	4129	4129	4279	4279

***p < 0.001; **p < 0.01; *p < 0.05 | *SRMR* standardised root mean square residual

## Discussion

The results of the present study support the hypothesis that superior social cognitive abilities as measured in middle childhood (ages 5 and 7) predict fewer mental health problems from middle childhood through to late adolescence in the general youth population. In the context of the MCS surveys, we employed the term ‘superior’ social cognitive abilities to mean that children had established (a) false-belief understanding, as demonstrated through answering the SAT questions correctly, first at age 5 and again at 7 years, and (b) social competence skills that allowed them to navigate a demanding social interaction with an unfamiliar interviewer-assessor. Using latent growth modelling, we found that these social cognitive abilities predicted decreasing trajectories (negative slope) of emotional problems over time. This association persisted even after controlling for sex, ethnicity, parental education, time-dependent family income across sweeps, and verbal ability at baseline.

The hypothesis was drawn from the evidence about the role of deficits in ToM and social cognition in youth psychiatric conditions. Here we wanted to understand whether those who had established superior social cognitive skills in middle childhood may be ‘protected’ from internalising and externalising problems later on. Our results suggest that this was indeed the case, at least with respect to emotional symptoms. Furthermore, for male cohort members, we found that conduct problems for those in the superior social cognitive abilities group were lower at baseline. For female cohort members in this group, the trajectory of conduct problems was found to be decreasing over time.

These findings extend previous work, which has linked deficits in social cognition with particularly conduct problems [56, 75]. They also extend previous finding showing that both internalising and externalising problems are linked with impairments in the broader construct of social competence. For example, testing developmental cascades in a sample of 117 children, Bornstein et al. [10] provided evidence that less socially competent children at age 4 years exhibit more externalising and internalising problems at age 10 years and more externalising problems at age 14 years, even after controlling for intelligence and maternal education. Our study contributes to this evidence by showing links of superior ToM with mental health across development in the general population. It would appear that superior social cognitive abilities permit a more skilful navigation of the social worlds in which children and adolescents find themselves in, thus protecting against emotional and behavioural problems [3, 8, 21].

Our study has several limitations. First, it is correlational, so we cannot determine whether the association between social cognitive abilities and mental health symptom trajectories is causal and not due to residual confounding. Second, our measure for ToM was based on a single false-belief task (SAT). Our analysis did not examine other false-belief tasks, or indeed other ToM measures [7]. Crucially, the SAT was not administered in an enacted storytelling format but relied on a vignette delivered in demanding dyadic interaction between the child and an unfamiliar interviewer-assessor. Third, we controlled for cognitive (verbal) ability in addition to maternal education, income, and demographic variables, but not a measure of executive function as a confounder at age 5 or 7 years, as this was not available in MCS in those survey sweeps. Fourth, the trajectories of mental health symptom scores were tracked through parent-reported SDQ scales; ideally, these would be complemented with teacher and self-reports and assessments by mental health professionals. Nonetheless, the present study also has significant strengths, including the use of data from a large and nationally representative UK birth cohort and the longitudinal recording of both our key measures (SAT and SDQ). The survey also allowed us to consider a variety of potential confounders at both family and child levels. Additionally, we were able to use four survey sweeps, in which the young person’s internalising and externalising problems were consistently tracked across 10 years and for a period that includes two important transitions, to puberty and secondary school.

The significant association between superior social cognitive skills in middle childhood and decreasing emotional symptoms over time suggests the possibility of impactful early interventions [38]. Social cognition and ToM abilities can be supported from an early age, both at home and in educational settings, through the use of mirroring and imitation [50, 59], eye contact [26], joint attention [81], mental state talk [69], and pretend play (Charman, 2000; [44]. Early years and primary school curricula can be expanded to include more of these activities.

## Data Availability

The data that support the findings of this study are available from the Millennium Cohort Study, UK Data Service of the University of Essex, University of Manchester and Jisc (https://ukdataservice.ac.uk/). The dataset is available from the UK Data Service by application, under licence.
